# The Need for “Health Twitteracy” in a Postfactual World

**DOI:** 10.3928/24748307-20170502-01

**Published:** 2017-06-14

**Authors:** Kristine Sørensen

Social media is increasingly playing a critical role for direct communication. One such example is Twitter, which is an online social media networking and microblogging service that enables users to send and read short 140-character messages called “tweets.” Registered users can read and post tweets, but unregistered users can only read them. The conversations on Twitter are often formed around hashtags (#) that can enable connections between people, foster the effective sharing of ideas, and help entire online communities form and flourish. As the fastest growing social media site (McCue, 2013), Twitter allows, for instance, organizations to communicate with a target audience and many other people by posting short messages without a significant amount of effort. The built-in mechanism with regards to the concise format and limited length pushes toward simplifying messages to the benefit of users; however, there are also pitfalls. At times, tweets can be impossible to understand because of jargon and abbreviations; tweets can also be misleading and dangerous and potentially drown everyone in a deluge of information. Yet, if building on principles of clear communication, tweets can also be meaningful and informative and emerge as important topics that become trends through retweeting and thus facilitate the flow of information by virtue of a dynamic and evolving ecology of networks (Park, Rodgers, & Stemmle, 2013).

## Health Literacy

**Figure 1.**
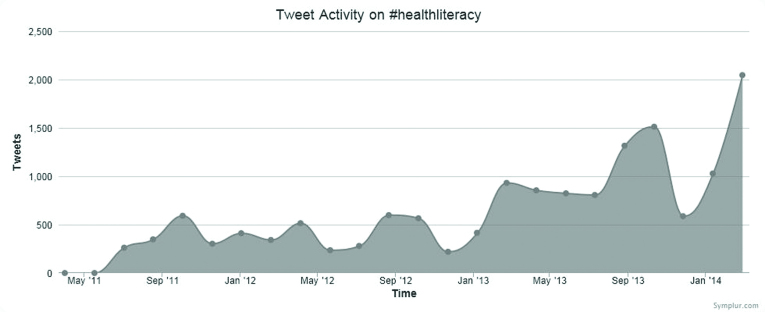
Distribution of #healthliteracy tweets between April 1, 2011 and April 15, 2014. Created from data on Symplur.com.

The health literacy community is an example of such a dynamic and evolving ecology of networks that has evolved in recent years. Health literacy is regarded as an essential skill for the 21st century (Kickbusch, 2008), and it can be defined as the knowledge, motivation, and competency to access, understand, appraise, and apply information to form judgment and make decisions concerning health care, disease prevention, and health promotion to maintain and improve quality of life during one's lifespan (Sorensen et al., 2012). To illustrate the evolution of the health literacy community on Twitter, data related to #healthliteracy was extracted through Symplur. Symplur hosts the Healthcare Hashtag Project, where health care–related hashtags are archived and can be searched and their associated tweets downloaded for analysis. The Healthcare Hashtag Project is a free open platform for patients, caregivers, advocates, doctors, and other providers that connects them to relevant conversations and communities (Symplur, 2017). According to Symplur, the first tweet including #healthliteracy was tweeted on May 11, 2011, and the number of tweets has been steadily growing since as illustrated in **Figure 1** (Symplur, 2014). A recent sample covering the month of February 2017 indicated that 1,482 tweets were sent by 767 participants reaching out to more than 3 million impressions (Symplur, 2017). Impressions pertain to the actual interaction or engagement after a tweet has been delivered to various Twitter streams. Hence, “tweet count” refers to the total number of tweets sent by an account, and the impression refers to the tweets sent that actually generate interaction or replies from others online. Notably, not all tweets or hashtags generate responses or trigger engagement (Doctor, 2013).

The tweets concerning #healthliteracy are most frequently sent by nonprofit organizations and community groups and are often quoted and retweeted by other Twitter users. Content wise, the tweets on health literacy topics were predominantly about using simple language rather than complicated language (Park et al., 2013), which is a common theme for the health literacy discourse, especially in North America (Pleasant & Kuruvilla, 2008; Sorensen, 2013).

## Health Literacy Champions on Twitter

Notably, we are facing a new era of communication. Whereas previous shifts included the telephone, which enabled one-to-one communication possible; the television, which ensured broadcasting to millions of people; and the Internet, which gave us access to countless websites; social media and Twitter, in particular, represents a new wave. With social media, it is possible to select people to follow and to receive direct messages as part of mass communication and stages of communication that are partly public and transparent and partly private. Furthermore, a vast amount of communication takes place in networks and the communication is briefer. People can choose large networks with shorter, more to-the-point conversation, rather than smaller networks with longer conversations. Lastly, communication channels have opened radically, providing open channels to, for instance, world leaders. It means people are reachable in more ways than ever (Gordhamer, 2010). Twitter is an increasingly popular resource for information dissemination and health professionals, and organizations are encouraged to use it as a tool for sharing information (Hughes, 2016). Health literacy–related organizations and networks to follow include for instance @theIHLA (International Health Literacy Association), @HL_Europe_net (Health Literacy Europe), and @gwgiuhpehl (IUHPE Global Working Group on Health Literacy). Furthermore, many individual health literacy champions are active on Twitter; for example, Ilona Kickbusch: @IlonaKickbusch, R.V. Rikard: @rv_rikard, and Andrew Pleasant: @andrewpleasant. Discussions concerning health literacy can be created and followed by referring to specific hashtags such as #healthliteracy, #healthlit, #plainlanguage, #PatientEngagement, and this journal—#HLRP (*HLRP: Health Literacy Research and Practice*).

## Health Twiplomacy

In recent months, we have experienced how “twiplomacy” has become of utmost importance in the political landscape. Twiplomacy refers to the use of Twitter and other social media sites by government agencies and officials to engage with the public, disperse information, and even leverage global influence. The term was coined by Burson-Marsteller, who studied world leaders on Twitter and attempted to illustrate how social media is closing the gap between these leaders and the public they serve (Techopedia, n.d.-a). For instance, the newly elected United States President Donald Trump is almost solely communicating with the population through Twitter. Statements posted on Twitter directed to his followers have a wide-reaching impact and the content is being discussed not only by users, but also by nonusers, because they are being repeated by other kinds of communication channels such as newspapers and television channels. The tremendous use of Twitter by President Trump along with the idea of “alternative facts” presented by an official US government spokesperson, raises questions worldwide on how to deal with this new way of communication in the political arena in the US and beyond.

## Health Twitteracy

Twitter, as a ground-breaking microblogging site, can be regarded as a powerful tool for democracy (e.g., citizen uprisings in the Middle East [Huang, 2011]) and disaster management and fundraising (e.g., earthquake in Italy and floods in Haiti [BBC, 2010]). Yet, it also has a flip-side. Due to Twitter's real-time flow, the news can spread faster than ever, whether trustworthy or not. Twitter's ‘thought leaders’ shape the agenda, whether evidence-based or not. The brevity of messages can also induce misunderstandings because of the oversimplified way of communication (Gross, 2011). Living in a postfactual world, we may ask ourselves if we, indeed, need a new category for health literacy, called “health twitteracy” to equip people with knowledge and competency to find, understand, appraise, and apply information from Twitter and other social media that they can trust and rely on to form judgements and make decisions to manage health for themselves and for their communities.

An example could be when new health care reforms are discussed regarding the impact of the shift from “Obamacare” to “Trumpcare.” Along these lines, research suggests that users searching for health information on Twitter would need certain literacy skills to interpret it correctly (Hughes, 2016), and tweeting, in particular, has been identified as a new literacy practice, comprising both traditional and new literacies and impacting both informal and formal learning settings (Greenhow & Gleason, 2012).

When Twitter received an award for societal impact in 2013, it was stated that “Twitter is the pulse of the planet” (Olanoff, 2013). Hence, when embracing the new era of communication, we need to be aware of Twitter as an important vital sign for communication. As knowledge brokers, health professionals play an essential role in providing timely, transparent, and correct health information, making it understandable, facilitating informed decision-making, and guiding people in how to use it to improve and maintain quality of life (Sorensen & Brand, 2011). Due to the social gradient of health literacy we know that people with less education and low socio-economic status have higher risks of facing problems when it comes to understanding the complexity of health and health systems (Berkman, Sheridan, Donahue, Halpern, & Crotty, 2011) and it will require extra effort to bridge the gap of lack of knowledge, motivation, competency, and resources. Recognizing that health literacy is closely associated to social justice and empowerment (Kickbusch, 2007), it is of utmost importance to invest in people's functional, interactive, and critical health literacy (Nutbeam, 2000) to facilitate a strong responsiveness toward the dissemination of fake news and misinterpreted evidence. This is especially important with regards to misinformation concerning health and healthy living, which could otherwise influence people's lives detrimentally. Indeed, following someone on Twitter does not mean that you necessarily endorse his or her tweets or agree with the person's perspective. Nevertheless, it allows you to be a part of the modern discourse being established in the Twitterverse (Techopedia, n.d.-b). Hence, when public health advocates fail to engage on Twitter, subsequently, they might yield power to tweeters who may or may not take a scientific and evidence-based approach to health.

Taking the recent developments in the policy arena into account, there is no doubt that health literacy, in particular, health twitteracy and eHealth literacy, defined as the ability to seek, find, understand, and appraise health information from electronic sources and apply the knowledge gained to addressing or solving a health problem (Norman & Skinner, 2006), will be required sooner rather than later.
